# Awareness of ovarian cancer symptoms and risk factors in a young ethnically diverse British population

**DOI:** 10.1002/cam4.5670

**Published:** 2023-02-07

**Authors:** Cristina‐Alexandra Radu, Nadia Matos de Melo Fernandes, Sumaira Khalfe, Britta Stordal

**Affiliations:** ^1^ Department of Natural Sciences Middlesex University London London UK

**Keywords:** awareness, ovarian cancer, risk factors, symptoms

## Abstract

**Background:**

Ovarian cancer does not cause many symptoms in the early stages, which is why the majority of cases are of advanced disease. Increasing awareness of ovarian cancer symptoms may lead to earlier diagnosis and improved outcomes.

**Methods:**

Participants in Britain completed the Ovarian Cancer Awareness Measure by online survey (*n* = 459).

**Results:**

Our participants were 75% female, 25% male and a young (27.89 ± 11.44 years) ethnically diverse population (40.3% White, 29.3% Asian and 18.0% Black). Individuals recalled 1.24 ± 1.30 symptoms, and recognised 5.96 ± 2.4 symptoms. We found higher levels of recall and recognition compared to previous research possibly due to using an online survey. Recognition was lowest for difficulty eating (39.4%) and persistently feeling full (38.7%). Males had slightly lower symptom recall and recognition than females. Participants incorrectly recalled an irregular menstrual cycle (22.4%) as an ovarian cancer symptom and 67% answered the age of incidence question incorrectly. Suggesting that participants incorrectly associate ovarian cancer as a disease of pre‐menopausal women.

Individuals recalled 1.47 ± 1.20 risk factors, and recognised 6.1 ± 2.4 risk factors. Family history of ovarian cancer was recalled by 59% of participants. Recognition was lowest for in vitro fertilisation treatment (23.0%) and talcum powder in the genital area (23.0%). The generic cancer risk factors of alcohol (9.3%) and poor diet (8.8%) were recalled as specific ovarian cancer risk factors. 57.9% of participants incorrectly answered that there is an ovarian cancer screening programme. Suggesting confusion between ovarian and cervical cancer as participants also recalled cervical cancer risk factors of sexually transmitted diseases (6.3%) and human papillomavirus (1.5%). 29.7% of female participants would seek help for an ovarian cancer symptom within 1–2 days. Help seeking was higher in the Black and Asian ethnicities (44.4% and 45.0%; *p* = 0.018).

**Conclusion:**

Awareness of ovarian cancer symptoms is low. Ovarian cancer awareness campaigns should include common misconceptions identified in this research.

## Abstract 2

Awareness of ovarian cancer symptoms is low. Participants incorrectly associate ovarian cancer as a disease of pre‐menopausal women as well as confusing it with cervical cancer. Ovarian cancer awareness campaigns should include common misconceptions identified in this research.

## 1 INTRODUCTION

Worldwide, there were 313,959 new cases of ovarian cancer in 2020, accounting for 3.4% of cancers diagnosed in women.^1^

Ovarian cancer is difficult to diagnose as the symptoms are non‐specific and can be confused with more common conditions such as irritable bowel syndrome.^2^ A general practitioner (GP) with an average sized practice may only see one case of ovarian cancer every 5 years.^3^ The symptoms that appear in the late stages are due to the intra‐abdominal pressure produced by the growing tumour.^4^ Bloating, increased abdominal size and urinary symptoms were found to co‐occur in 43% of women with ovarian cancer.^5^ Women with ovarian cancer experience symptoms 20–30 times per month with higher severity than women with benign masses or controls.^5^

There is currently no population screening programme for ovarian cancer. Early detection of ovarian cancer through identification of early symptoms is therefore critical to patient outcome. In England, 5‐year survival for ovarian cancer is 93.3%^6^ when diagnosed at FIGO Stage 1, when the cancer is limited to the ovaries or fallopian tubes.^7^ In contrast, when diagnosed at FIGO Stage 3, when the cancer has spread outside the pelvis,^7^ the 5‐year survival is 26.9%.^6^ Current guidance is that if a woman, particularly if over 50, reports any of the following symptoms—persistent bloating, pelvic or abdominal pain, increased urinary urgency or feeling full/loss of appetite—they be referred for serum CA125 testing.^8^ If serum CA125 is ≥35 IU/mL, they are referred for an ultrasound of the abdomen and pelvis.^8^ Raising awareness of ovarian cancer symptoms, so women can have informed conversations with their GP, is critical in achieving an earlier diagnosis and therefore increasing survival.

The lifetime risk of ovarian cancer for a woman in the UK is 2%.^9^, ^10^, ^11^, ^12^, ^13^, ^14^ The risk of ovarian cancer increases with an increase in the number of ovulatory cycles in a woman's lifetime.^15^ Which is why nulliparity^16^ increases the risk of ovarian cancer and pregnancy, breastfeeding^17^, ^18^ and use of the contraceptive pill^19^ all decrease the risk of ovarian cancer.

In this study, participants in the UK completed the Ovarian Cancer Awareness Measure^20^ by online survey. Our study provides a unique insight, as it is the first to collect data on ovarian cancer symptom and risk factor awareness from men as well as women. Our study also reflects the ethnic diversity of the London population.^21^ This study will provide a baseline of knowledge in these understudied groups to inform ovarian cancer education awareness campaigns.

## 2 METHODS

### 2.1 Survey

Participants completed the Ovarian Cancer CAM^20^ using the online survey tool Qualtrics.^22^ The Ovarian Cancer CAM is available from Cancer Research UK.^23^

### 2.2 Participants

Participants were recruited through the social media networks of Biomedical Science dissertation students and staff at Middlesex University. Some Biomedical Science and Biology students completed the survey as an in‐class activity before a lecture on ovarian cancer. Participants were recruited between November 2015 and June 2022.

The project received ethics approval from the Natural Science Research Ethics Committee at Middlesex University London. Students participating in the project were aware that it was for research and it was not a requirement for assessment or course credit. In the online survey, participants did not sign informed consent. However, the front page of the survey included a participant's information sheet, clicking into the survey acknowledged consent and participants were free to close the survey at any time and not participate. At the end of the survey, participants were given web links to the charity Target Ovarian Cancer if they wanted further information on ovarian cancer.

### 2.3 Analysis of recalled symptoms and risk factors

Participants had a free‐text response to recall their knowledge of ovarian cancer symptoms or risk factors. These free‐text responses were scored manually against the list of correct symptoms and risk factors on the Ovarian Cancer CAM.^20^ Symptoms included in the Ovarian Cancer CAM^20^ are those that have been associated with epithelial ovarian cancer, and not cancer in general. Risk factors are those which increase the risk of the disease occurring, even if some of the included only have a modest association. Protective factors for reducing the risk of ovarian cancer were not included in this list. Table S1 lists the range of answers that were scored correctly for each symptom and risk factor. The most frequent incorrect answers were also analysed. Table S2 lists the range of answers that were included for each incorrect symptom or risk factor.

### 2.4 Analysis of recognised symptoms and risk factors

Participants were given a list of known ovarian cancer symptoms or risk factors and asked if they thought they were associated with the disease. Participants were given a mark for each symptom or risk factor they recognised.

### 2.5 Age of incidence

Knowledge of the age of ovarian cancer incidence was assessed with a multiple‐choice question of age ranges. This was a modification to the ovarian cancer CAM which uses a specific age^23^ Participants were marked correct if they chose either 50–69 years or over 70 years, reflecting the highest incidence of ovarian cancer in the post‐menopausal population.

### 2.6 Cancer diagnosis or experience

Participants were asked if they had been previously diagnosed with cancer in a multiple‐choice question. Participants were categorised has having a personal experience with cancer if they reported their partner, close family member or a friend having cancer.

### 2.7 Demographic groups

Participants, who answered other or prefer not to say for a question, were excluded from the analysis of that individual question. The ethnicity question on the survey had 17 options for different ethnic groups as used in the UK census.^21^ These groups were collapsed into White, Asian, Black and Mixed as shown in Table S3. The mixed ethnicity group was not used for analysis due to low numbers of participants. The education question was dichotomised into Completed University‐Level Education and Completed Secondary‐School Education. Only one participant answered no formal education and they were excluded from the analysis for that question only.

### 2.8 Postcode analysis

Participants who provided their postcode data were mapped visually using Google Maps^24^ using the London Region boundary from the Office of National Statistics.^21^ Each postcode was matched to an LSOA code and participants were classified as living in London if they resided in the London Boroughs (City of London, Barking and Dagenham, Barnet, Bexley, Brent, Bromley, Camden, Croydon, Ealing, Enfield, Greenwich, Hackney, Hammersmith and Fulham, Haringey, Harrow, Havering, Hillingdon, Hounslow, Islington, Kensington and Chelsea, Kingston upon Thames, Lambeth, Lewisham, Merton, Newham, Redbridge, Richmond upon Thames, Southwark, Sutton, Tower Hamlets, Waltham Forest, Wandsworth, Westminster).^25^ Participants who provided their postcode data and resided in England (*n* = 355) were categorised as residing in areas of relatively high (Decile 1–5) or low (Decile 6–10) deprivation based on the English Indices of Deprivation 2019.^26^

### 2.9 Statistical analysis

Statistical analysis was performed using Minitab.^27^ Recall and Recognition figures were analysed across demographic groups using either the Mann–Whitney test (two‐group comparison) or Kruskal–Wallis test (three groups) as the data were not normally distributed. Confidence in observing an ovarian cancer symptom, time to help seeking, knowledge of screening and age of incidence were all analysed across demographic groups using the chi‐squared test. *p* < 0.05 was considered significant. Graphs were made using Graphpad Prism.^28^

### 2.10 Lifetime risk of ovarian cancer

It was calculated using UK female ovarian cancer incidence^9^ and mortality data^10^ 2016–2018 with UK female population^12^ and death data^13^ using Cancer Research UK's lifetime risk calculator^14^ adjusting for multiple primaries.^11^

## 3 RESULTS

### 3.1 Participants

In all, 449 participants responded to the survey. The gender balance was 75.1% female and 24.1% male. The mean age of participants was 27.89 years and the range was 18–75 years. The ethnic diversity of the participants was 40.3% White, 29.3% Asian and 18% Black (Table 1).

**TABLE 1:** Study participants.

		All participants (*n* = 449)	Age < 30	Age ≥ 30	*p*‐Value Chi squared vs age groups
Age (*n* = 420)		Mean 27.89 ± 11.44 Range 18–75 years	312 (74.2%)	108 (25.7%)	
Gender (*n* = 449)	Female	337 (75.1%)	231 (73.6%)	92 (85.2%)	0.045
	Male	108 (24.1%)	80 (25.5%)	15 (13.9%)	
	Prefer not to say	4 (0.8%)	3 (1.0%)	1 (0.9%)	
Ethnicity (*n* = 427)	White	172 (40.3%)	84 (26.8%)	85 (78.7%)	0.000
	Asian/Asian British	125 (29.3%)	114 (36.4%)	10 (9.3%)	
	Black/African/Caribbean/Black British	77 (18.0%)	70 (22.4%)	6 (5.6%)	
	Mixed/Multiple Ethnic Groups	21 (4.9%)	16 (5.1%)	4 (3.7%)	
	Other	26 (6.1%)	23 (7.3%)	3 (2.8%)	
	Prefer not to say	6 (1.4%)	6 (1.9%)	0 (0.0%)	
Postcode (*n* = 355)	London Region	276 (77.7%)	229 (84.5%)	47 (56.0%)	0.000
	Non‐London Region	79 (22.3%)	42 (15.5%)	37 (44.0%)	
Deprivation (*n* = 355)	High Deprivation (Decile 1–5)	222 (62.5%)	191 (70.5%)	31 (36.9%)	0.000
	Low Deprivation (Decile 6–10)	133 (37.5%)	80 (29.5%)	53 (63.1%)	
Education (*n* = 421)	Completed university education	257 (62.0%)	165 (47.5%)	92 (85.2%)	0.000
	Completed school education	165 (35.2%)	149 (52.6%)	16 (14.8%)	
Cancer diagnosis (*n* = 412)	Cancer diagnosis	43 (10.4%)	11 (3.6%)	32 (30.5%)	0.000
	No cancer diagnosis	363 (88.1%)	291 (94.8%)	72 (68.6%)	
	Prefer not to say	6 (1.5%)	5 (1.5%)	1 (1.0%)	
Cancer experience (*n* = 416)	Personal experience of cancer	291 (70.0%)	193 (62.5%)	98 (91.6%)	0.000
	No experience of cancer	125 (30.0%)	116 (37.5%)	9 (8.4%)	

There were two distinct populations who responded to the survey. A younger ethnically diverse population (<30) was 73.6% female and 25% male. Participants over 30 were significantly less ethnically diverse and were primarily female (Table 1). The younger population was more likely to reside in London and in a more deprived area (Table 1). Therefore, all demographic comparisons were examined using the <30 cohort. The older cohort was only used for two analyses: (i) the comparison of age itself, and this was limited to the white cohort and (ii) the impact of a cancer diagnosis as numbers for this were limited in the young cohort. Comparisons between ethnicities were limited to Asian, Black and White participants due to lower numbers in the other groups.

### 3.2 Ovarian cancer symptom recall and recognition

**FIGURE 1:**
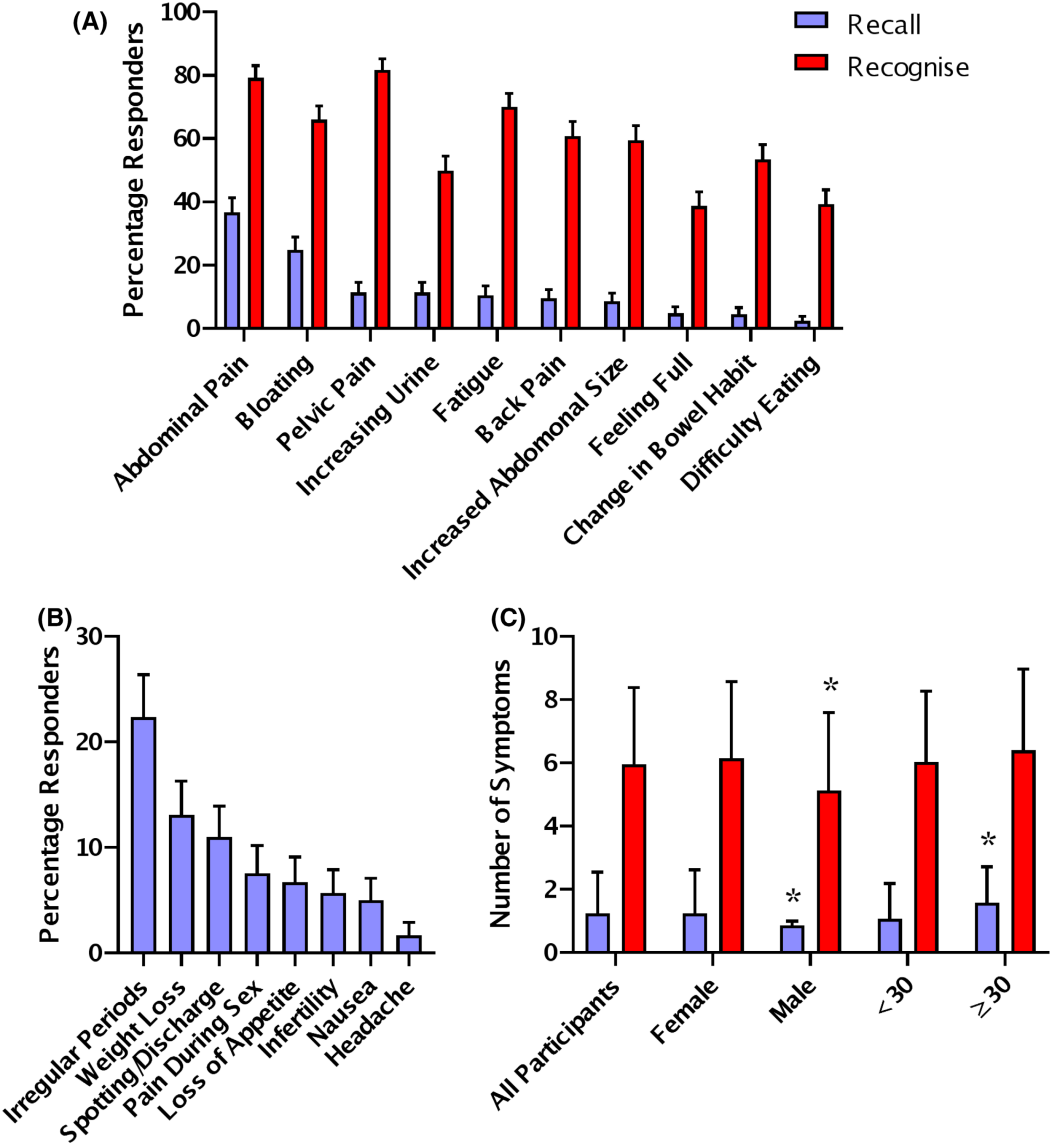
Ovarian cancer symptom recall and recognition. (A) Recall and recognition of ovarian cancer symptoms; percentage responders shown with 95% CI (Recall *n* = 420; Recognition *n* = 447). (B) Recall of incorrect ovarian cancer symptoms; percentage responders shown with 95% CI (*n* = 420). (C) Number of symptoms recalled and recognised per participant; mean and SD. All participants (*n* = 449), Female versus Male <30 * *p* < 0.05 Mann–Whitney Test (*n* = 338). Recall is shown in purple and recognition in red.

Participants recalled ovarian cancer symptoms unprompted by typing in a free‐text box, they recognised symptoms by choosing from a presented list. The most frequently recalled ovarian cancer symptoms were abdominal pain (36.7%) and bloating (24.8%) (Figure 1A). The least frequently recalled ovarian cancer symptoms were change in bowel habit (4.5%) and difficulty eating (2.4%) (Figure 1A). In general, the most frequently recalled symptoms were also the most frequently recognised, but there were some changes in the order. The most frequently recognised ovarian cancer symptoms were pelvic pain (81.7%) and abdominal pain (79.2%) (Figure 1A). The least frequently recognised ovarian cancer symptoms were difficulty eating (39.4%) and feeling full persistently (38.7%) (Figure 1A).

The most commonly recalled incorrect symptoms for ovarian cancer were irregular periods (20.9%) and weight loss (12.2%) (Figure 1B). 36.7% of participants could not recall any correct ovarian cancer symptoms. On average, each participant was able to recall 1.25 ± 1.3 symptoms and recognise 5.9 ± 2.4 symptoms. In the young cohort, males recalled slightly less symptoms and recognised one less symptom than females (*p* = 0.003 and *p* = 0.003, Mann–Whitney Test). There was no difference in the recall or recognition of symptoms based on ethnicity, deprivation university education or experience with cancer in the young cohort. Participants over 30 recalled slightly more symptoms than younger participants (*p* = 0.003, Mann–Whitney Test) (Figure 1C). In the older cohort, there was no difference in symptom recall or recognition associated with a previous cancer diagnosis.

### 3.3 Ovarian cancer risk factor recall and recognition

**FIGURE 2:**
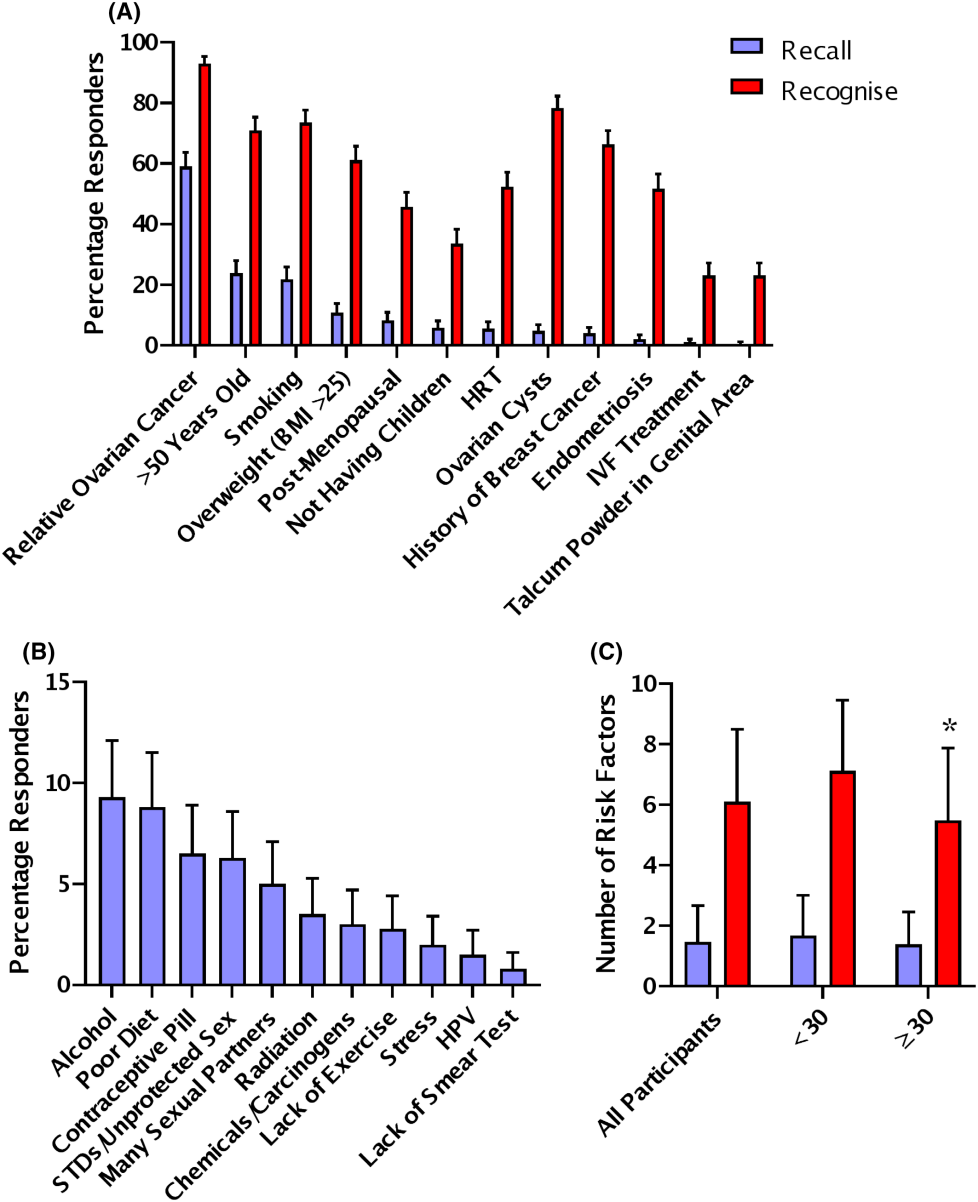
Ovarian cancer risk factor recall and recognition. (A) Recall and recognition of ovarian cancer risk factors; percentage responders shown with 95% CI (Recall *n* = 400; Recognition *n* = 429). (B) Recall of incorrect ovarian cancer risk factors (*n* = 400). (C) Number of risk factors recalled and recognised per participant; mean and SD. All participants (*n* = 400), Age < 30 versus > 30 White participants * *p* < 0.05 Mann–Whitney Test (*n* = 154). Recall is shown in purple and recognition in red.

Participants recalled ovarian cancer risk factors unprompted by typing in a free‐text box, they recognised risk factors by choosing from a presented list. The most frequently recalled ovarian cancer risk factors were a family history of ovarian cancer (59.0%) and older age (23.8%) (Figure 2A). The least frequently recalled ovarian cancer risk factors were in vitro fertilisation (IVF) treatment (1.0%) and use of talcum powder in the genital area (0.5%) (Figure 2A). In general, the most frequently recalled symptoms were also the most frequently recognised, but there were some changes in the order. The most frequently recognised ovarian cancer risk factors were family history (93.0%) and smoking (73.5%) (Figure 2A). The least frequently recognised ovarian cancer symptoms were IVF treatment (23.0%) and use of talcum powder in the genital area (23.0%) (Figure 2A).

The most commonly recalled incorrect risk factors for ovarian cancer were alcohol use (9.3%) and poor diet (8.8%) (Figure 2B). 19.5% of participants could not recall any correct ovarian cancer risk factors. On average, each participant was able to recall 1.47 ± 1.20 factors and recognise 6.1 ± 2.4 risk factors. In the young cohort, there was no difference in the recall or recognition of risk factors based on gender, ethnicity, deprivation, university education or experience with cancer. Participants over 30 recognised 1.5 fewer risk factors than younger participants (Figure 2C). In the older cohort, there was no difference in risk factor recall or recognition associated with a previous cancer diagnosis.

### 3.4 Knowledge of ovarian cancer screening and age of incidence

**FIGURE 3:**
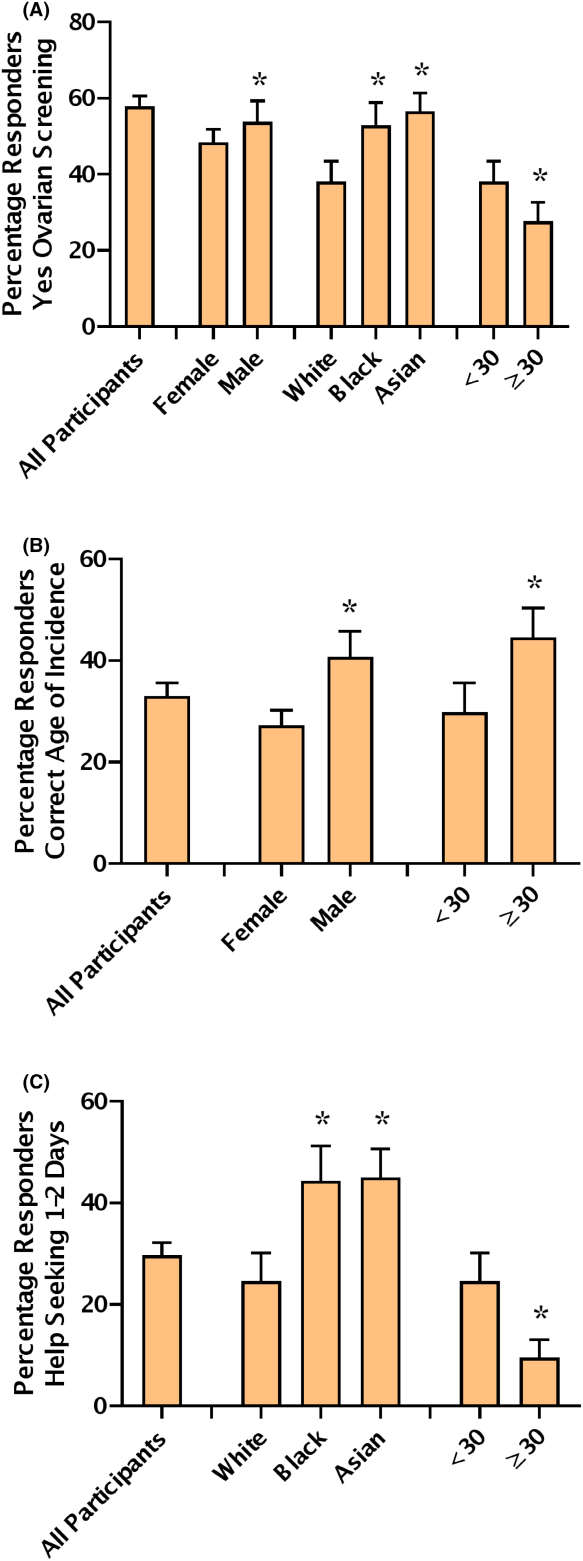
Knowledge of ovarian cancer screening, age of incidence and time to help seeking. (A) Participants incorrectly answering that there is an NHS ovarian screening programme; percentage responders shown with 95% CI. All participants (*n* = 432), Female versus Male <30 (*n* = 309), Ethnicity White versus Black or Asian <30 (*n* = 267), Age < 30 versus > 30 White participants (*n* = 167). (B) Participants correctly answering the age of incidence question; percentage responders shown with 95% CI. All participants (*n* = 432), Female versus Male <30 (*n* = 309), Age < 30 versus > 30 White participants (*n* = 167). (C) Female participants responding a fast Time to Help Seeking; percentage responders shown with 95% CI. All female participants (*n* = 337), Ethnicity White versus Black or Asian <30 (*n* = 195), Age < 30 versus > 30 White participants (*n* = 134). * *p* < 0.05 chi‐squared test.

57.9% of participants incorrectly answered that there was an NHS ovarian cancer screening program (Figure 3A). In the young cohort, more males and participants from Black and Asian ethnicity answered the screening question incorrectly (*p* = 0.20 and *p* = 0.028, chi‐squared test, Figure 3A). In the young cohort, there was no difference answering the screening question based on deprivation, university education or experience with cancer. Older participants were also more likely to answer the question correctly (*p* = 0.004, chi‐squared test, Figure 3A). In the older cohort, there was no difference in knowledge of screening associated with a previous cancer diagnosis.

Only a third of participants answered the age of ovarian cancer incidence question correctly. In the young cohort, males were more likely to answer the age of incidence correctly (*p* = 0.019, chi‐squared test, Figure 3B). In the young cohort, there was no difference answering the age of incidence question based on ethnicity, deprivation university education or cancer experience. Participants over 30 were more likely to answer the age of incidence correctly (*p* = 0.048, chi‐squared test, Figure 3B). In the older cohort, there was no difference in the age of incidence question associated with a previous cancer diagnosis.

### 3.5 Time to help seeking

29.7% of female participants answered that they would seek help for an ovarian cancer symptom within 1–2 days (Figure 3C). In the young cohort, help seeking was substantially higher in the Black and Asian ethnicities with 44.4% and 45.0% answering that they would seek help in 1–2 days (*p* = 0.018, chi‐squared test, Figure 3C). In the young cohort, there was no difference answering the help‐seeking question based on deprivation, university education or experience with cancer. In the young cohort, those who reported the fastest time to help seeking of 1–2 days, recognised less ovarian cancer symptoms than those who would seek help later (5.48 ± 2.28; *p* = 0.029, Kruskal–Wallis Test).

Help seeking was lower in the older age group with only 9.6% of female responders over 30 answering that they would seek help quickly (*p* = 0.000, chi‐squared test, Figure 3C). In the older cohort, time to help seeking tended to be slower in those with a previous cancer diagnosis but this was not significant (*p* = 0.081, chi‐squared test). In the older cohort, help seeking was not associated with symptom or risk factor recall or recognition.

### 3.6 Confidence in recognising an ovarian cancer symptom

**FIGURE 4:**
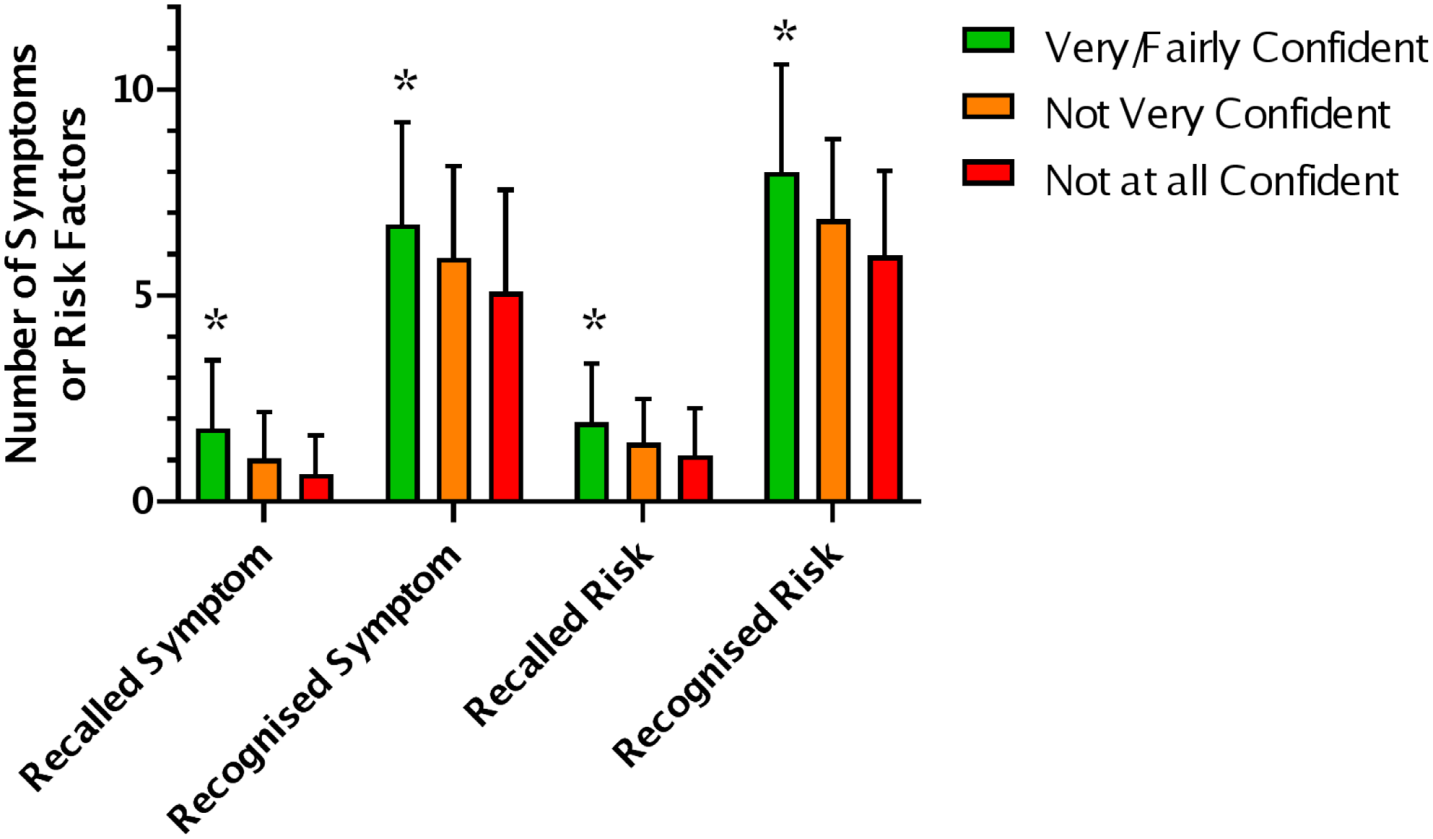
Participants confidence and recall and recognition of symptoms and risk factors <30. Number of symptoms recalled and recognised per participant; mean and SD. Symptom Recall (*n* = 291), Symptom Recognition (*n* = 310), Risk Factor Recall (*n* = 289) and Risk Factor Recognition (*n* = 308) * *p* < 0.05 Kruskal–Wallis Test. Very/Fairly confident shown in green, Not very confident in orange and Not at all confident in red.

Confidence in recognising an ovarian cancer symptom was low with only 1.4% very confident and 22.6% fairly confident. In the young cohort, males tended to be less confident than females but the difference was not significant (*p* = 0.063 chi‐squared test). No difference in confidence was found based on ethnicity, deprivation, university education or experience with cancer in the young cohort. In the young cohort, confidence (very or fairly confident) was associated with higher symptom and risk factor recall and recognition (Figure 4A; *p* = <0.05, Kruskal–Wallis Test). There is a trend for the same pattern in the older cohort, but it is not significant due smaller sample size. There was no difference in confidence between the young and old cohorts.

### 3.7 Online survey quality control

The median time to complete the online survey was 8.7 min. 17.8% of participants took longer than 15 min. There was no significant difference in the number of symptoms or risk factors recalled or recognised by participants who took longer to complete the survey. There was also no difference in knowledge of the lack of ovarian cancer screening or age of incidence. This suggests that participants did not look up the answers if they took longer to complete the online survey.

41.6% of participants completed the Ovarian Cancer CAM as an in‐class activity as part of their Biomedical Science or Biology degree at Middlesex University. Some differences were seen in the in‐class group compared to those who completed it from an email or social media link. The in‐class group were more likely to get the screening question incorrect (61%), as well as recall fewer ovarian cancer symptoms (1.05 ± 1.11). However, the in‐class group was more likely to be male (33.6%) and younger (22 ± 2.88) and this follows the same pattern we see in the previous analysis.

Data were collected from November 2015 to June 2022. Some differences were seen based on year of recruitment. However, this does not reflect a change in ovarian cancer awareness over time but rather the demographics of participants based on different recruitment techniques. From 2015 to 2016, 160 participants were recruited via email/social media with an average age of 29.4 ± 11.4. From 2019 to 2020, 187 participants were recruited primarily through in‐class activities, with an average age of 21.9 ± 2.9. In 2022, 71 participants were recruited via email/social media deliberately targeting an older age group to balance the dataset with an average age of 40 ± 14.7.

In all, 18 participants (3.9%) were determined to share an IP address with another respondent. Analysis of the demographic answers suggests that these are 18 unique participants. The answers did not improve on a subsequent submission from the same IP address. It is likely that family units were responding to the survey on the same device for dissertation students.

## 4 DISCUSSION

### 4.1 Ovarian cancer symptom awareness may be improving in the UK

The UK charity Target Ovarian Cancer has been regularly campaigning about the symptoms of ovarian cancer^29^ since the Low et al's study in 2013 which they funded.^4^ In 2020, there was a NHS England's Help Us Help You campaign focusing on abdominal and urological symptoms of cancer.^30^ Target Ovarian Cancer's most recent pathfinder study shows an increased awareness of bloating and pelvic or abdominal pain in 2022 compared to their first study in 2009.^31^

In our study, recall of ovarian cancer symptoms is in general low, but is higher in this study than previous studies in the UK.^4^, ^20^ In our study, 36.7% of participants could not recall any symptoms and the average recall was 1.25 ± 1.3 symptoms per participant (Figure 1C). This is an improvement on the 2013 study by Low et al. where 58% of participants were unable to recall any symptoms and the average symptom recall was only 0.6 ± 0.8 symptoms per participant.^4^ This is also an improvement on the Simon et al. 2012 validation study for the ovarian cancer CAM where the average symptom recall was 0.8 ± 0.7 symptoms per participant.^20^ However, the methodological differences between studies should be noted. Low et al.'s study was a nationally representative sample.^4^ Whereas our study and Simon et al.'s study (2012) had similar opportunistic recruitment among students and staff at a university and their relatives.^20^

A similar pattern of symptom recall is seen in this study compared to the literature. Recall of abdominal or pelvic pain and bloating is the highest frequency, whereas feeling full persistently and difficulty eating the lowest.^4^ In our study, 12.7% more participants recalled abdominal pain and 15.8% more recalled bloating than Low et al.'s study (Figure 1A).^4^

The ovarian cancer CAM was delivered as an online survey in this study, whereas the Low et al.'s study was conducted by telephone survey.^4^ Comparisons of surveys of knowledge of scientific facts conducted online versus telephone surveys have found that an online survey produced less item non‐response, and a higher percentage of correct answers.^32^ This may explain in part the slightly higher levels of symptom recall in this study compared to Low et al.'s study.^4^ Our participants were instructed not to look up the answers to the survey, but as it was unsupervised it is not possible to prevent some people consulting the internet. However, we do have evidence to suggest that the majority of participants did not consult the internet as 98% of participants either included an incorrect symptom or risk factor or answered the ovarian cancer screening or age of incidence questions incorrectly. Those participants who took longer than 15 min to complete the survey also did not have any more correct answers. A recent study by Cancer Research UK compared the general cancer awareness measure delivered online versus face to face. They found a similar increase in recall of symptoms to our study but concluded that online data collection was a viable method.^33^

### 4.2 Ovarian cancer symptom recognition is consistent with previous UK studies

Recognition of ovarian cancer symptoms is similar in this study to previous studies in the UK.^4^, ^34^ In our study, the average recognition was 5.9 ± 2.4 symptoms per participant (Figure 1C), which is similar to the average of 6.3–6.85 reported in other UK studies using the ovarian CAM.^4^, ^34^ A similar pattern of symptom recognition is seen in this study compared to the literature.^4^, ^34^ Recognition of abdominal or pelvic pain and bloating is the highest frequency, whereas feeling full persistently and difficulty eating the lowest.

### 4.3 Recall of ovarian cancer risk factors is higher than recall of ovarian cancer symptoms

Recall of ovarian cancer risk factors was 18% higher than ovarian cancer symptoms (*p* = 0.001; Mann–Whitney Test). Recall of the two highest risk factors, a family history of ovarian cancer (59.0%) and age (23.8%), were good, although less people linked older age to being post‐menopausal (8.35%) (Figure 2A). We could find no other UK studies examining the recall of ovarian cancer risk factors.

Of note is the low level of recall of the use of talcum powder in the genital area (0.5%) as a risk factor for ovarian cancer. The use of talcum powder in the genital area has been associated with an increased ovarian cancer risk in retrospective case–control studies.^35^ However, prospective studies following women over time have not found the same association.^36^, ^37^ Therefore, a lack of public awareness of a risk associated with talcum powder is not problematic as the association is controversial.

A similar pattern of risk factor recognition is seen in this study with the literature. In our study, 93.0% of participants recognised family history of ovarian cancer, and 66.4% recognised a past history of breast cancer. This is a higher level of recognition than seen in the Fallowfield 2010 study, 5.9% more for family history of ovarian cancer and 46.8% more for past history of breast cancer.^38^

The Fallowfield 2010 study was conducted on 21,715 post‐menopausal women in the UK with an age range of 50–74 years, where 14.8% had a university education.^38^ Within the Fallowfield post‐menopausal cohort, the youngest women were more likely to correctly recognise ovarian cancer risk factors.^38^ We see the same pattern in our study where our younger cohort, under 30 years, recognise more risk factors (Figure 2B). The Fallowfield study questionnaire was conducted by mail,^38^ the participants were part of a larger ovarian cancer screening trial.^39^ In contrast to telephone surveys, studies show little difference in the answers given in an online survey compared to a paper‐based questionnaire.^40^, ^41^

### 4.4 Incorrect symptoms and risk factors

The correct symptoms and risk factors were taken from the original Ovarian Cancer Awareness Measure.^20^ This study was informed by the literature at the time of development as well as expert opinion advising the project. The inclusion or exclusion of individual symptoms or risk factors are of course open for debate. Weight loss for example, which is a symptom of many cancers and is included on the general cancer awareness measure,^23^ was not included as a correct symptom as it has been shown not to be characteristic of women diagnosed with ovarian cancer compared to other women attending primary care clinics.^5^ Unintentional weight loss (cachexia) does occur, particularly in advanced ovarian cancer.^42^ However, it has not been shown to be useful in differential diagnosis compared to other more frequent symptoms such as bloating and pelvic pain.

22.4% of participants suggested that irregular periods were a symptom of ovarian cancer and 5.7% suggested infertility (Figure 1B). These incorrect symptoms demonstrate that participants consider ovarian cancer to be a disease largely affecting younger pre‐menopausal women. Only a third of participants correctly answered that the highest incidence of ovarian cancer was in post‐menopausal women (Figure 3B). Of the participants who suggested irregular periods or infertility as an ovarian cancer symptom, 78% and 87% answered the age of incidence question incorrectly.

Irregular periods are not a symptom of epithelial ovarian cancer. However, irregular periods are associated with rarer non‐epithelial ovarian cancer subtypes such as granulosa cell tumours.^43^, ^44^ Irregular periods have been shown to be protective against developing ovarian cancer before age 45.^45^ In contrast, irregular periods are a risk factor for developing ovarian cancer over the age of 70.^46^ Infertility, while not a symptom of ovarian cancer can also lead to increased risk as nulliparity is a risk factor.^16^ IVF treatment may be a risk factor for ovarian cancer, and we and the ovarian cancer CAM have included it as a risk factor. However, a recent Cochrane review found that the majority of studies had methodological issues and needed longer follow‐up times.^47^ Some studies have found no increase in risk of ovarian cancer in women receiving IVF if they go on to give birth, but an increased risk in those who do not.^16^, ^48^ Essentially finding that nulliparity is a risk factor. Others have found an increased risk in women receiving IVF who gave birth.^49^

Many of the incorrect risk factors recalled by participants show that they quite reasonably associate an unhealthy lifestyle with cancer risk. Excessive alcohol consumption is associated with the risk of many cancers^50^, ^51^ but not ovarian cancer.^52^ Poor diet and lack of exercise were recalled by many participants which would contribute to a high BMI which is a risk factor for ovarian cancer in pre‐menopausal women.^53^

57.9% of participants in our study thought that there was an NHS ovarian cancer screening programme indicating confusion between cervical and ovarian cancer. This is consistent with the results of other studies where 40.1%–67% of women thought that a smear test could detect ovarian cancer.^54^, ^55^ Participants in this study also recalled many risk factors that are associated with the risk of cervical cancer and not ovarian cancer such as sexually transmitted diseases, a large number of sexual partners and human papillomavirus (HPV).^56^

The confusion between cervical and ovarian cancer could be used as a starting point for new education campaigns on gynaecological cancer. Rather than a campaign focusing on one cancer type, a campaign which explains that the HPV vaccine does not protect against all gynaecological cancers and alerting the public to key symptoms of cervical and ovarian cancer. A successful programme in the United States called Inside Knowledge: Get the Facts about Gynaecologic Cancer used a face‐to‐face information session approach increased women's knowledge that cervical screening does not screen for ovarian cancer and that genetic testing is available.^57^ In the UK, Target Ovarian Cancer's Pathfinder 2022 report has called for information to be provided at cervical screening appointments to make clear that cervical screening does not test or screen for other gynaecological cancers and include symptoms to look out for.^31^ Our research findings support this position.

### 4.5 Demographic factors

#### 4.5.1 Education and Socioeconomic Status

Our participants were more likely to have completed university education (62%) than the general London population (37.7%).^21^ We did not find a difference in knowledge based on completing university education in this study. One limitation is that only one participant answered that they had no qualifications compared to 17.6% of the London population.^21^

It is unclear if our participants are more highly educated than the previous studies examining symptom recall.^4^, ^20^ The 2012 validation study described their participants as more educated than the general population but did not provide details.^20^ The 2013 study used a simplified socioeconomic score (SES), based on having any formal education, car and home ownership, but did not report on education individually.^4^ 54% of the 2013 study were high SES indicating education as well as home and car ownership, the study found high SES predictive of more knowledge. We did not base our primary analysis on the SES score as it has been recommended for older retired participants.^4^, ^58^ We instead used the English Indices of Deprivation,^26^ and found no differences between participants from areas of high versus low deprivation. In order to compare with the 2013 study, we calculated the SES score for our participants. It became clear why it may not be suitable as while highly educated, our younger primarily London‐based population is more likely to live with family/friends (41.1%) than to own their own home (21.0%). 18.6% of our participants had a high SES score and this was significantly associated with age (*p* = 0.000; chi‐squared test). Some differences were seen in the high SES group, they were more likely to get the screening question correct (66.6%), as well as recall more ovarian cancer symptoms (1.70 ± 1.24) and recognise less risk factors (5.72 ± 2.34) (*p* = 0.001 and *p* = 0.001; Kruskal–Wallis Test). This follows the same pattern we see in the previous analysis on age of participants in Figures1C, 2C and 3A.

#### 4.5.2 Age and gender

Males recalled fewer ovarian cancer symptoms and risk factors than females (Figure 1C, 2C). The response of males has not been shown in an ovarian cancer awareness study before but is consistent with the wider cancer awareness literature.^59^ Our participants over the age of 30 recalled more symptoms but recognised less risk factors. Recall and recognition of general cancer warning signs has been shown to increase with age, peaking in the 55–64 age group and then declining in those aged over 65 years.^59^

#### 4.5.3 Ethnicity

Our participants were more ethnically diverse than the general London population which is 59.8% white.^21^ This allows our study to look into ethnicity in more detail than previous cancer awareness studies who combined non‐white participants due to low numbers.^4^, ^34^, ^59^ Help seeking was higher in the Black and Asian ethnicities (44.4% and 45.0%; *p* = 0.018). This effect has been observed previously in non‐White participants in the Low et al.'s UK ovarian cancer awareness study.^4^ In the UK, South‐Asian women have been shown to have a lower risk of ovarian cancer than white or black women.^60^ Despite this increase in help‐seeking in the Black and Asian ethnicities in the UK, ovarian cancer diagnosis is often later in non‐white populations such as in African‐Americans in the United States.^61^ Data are lacking on relative speed of diagnosis of ovarian cancer by ethnicity in the UK population.

Participants from Black and Asian ethnicities were also more likely to incorrectly believe that there was a screening programme for ovarian cancer (Figure 3A). However, the overlap between participants who had high‐help seeking as well as incorrect knowledge of screening was 55%, increasing to 60% in the Asian and Black ethnic groups. High levels of help seeking behaviour may therefore not translate into participation in national screening programmes; as some studies have shown that ethnic minority women are less likely to attend cervical screening in the UK, particularly if they were born overseas or from a South Asian background.^62^, ^63^ However, these are small studies and uptake of cervical screening data by ethnicity is not available from the NHS cervical screening programme.

### 4.6 Strengths and limitations

A limitation of this study is that it is not a population study designed to be representative of either the UK or London population. However, our opportunistic recruitment through the student population of Middlesex University was a strength of this study as it allowed us to examine a more ethnically diverse population. We show very little differences across demographic groups suggesting that there would be no benefit of a highly targeted campaign for a particular group. Another limitation is that the majority of our participants are not post‐menopausal women, who experience the highest incidence of ovarian cancer. Post‐menopausal women would be the primary group targeted by ovarian cancer awareness campaigns. However, the inclusion of men and a young population in general allows us to show that knowledge of ovarian cancer is low across this part of the population and that awareness campaigns would be of benefit to all.

## 5 CONCLUSIONS

Awareness of ovarian cancer symptoms is low and risk factors is good. This is the first UK study to include male participants and have high levels of ethnic diversity. Small differences in awareness are observed by gender and age. Our study demonstrates that delivering the ovarian cancer CAM by online survey is a viable research method. Ovarian cancer awareness campaigns should include common misconceptions such as a younger age of incidence and confusion with cervical cancer.

## ETHICS APPROVAL STATEMENT

This study was approved by the Department of Natural Sciences Research Ethics Committee at Middlesex University.

## AUTHOR CONTRIBUTIONS

**Cristina‐Alexandra Radu:** Formal analysis (supporting); project administration (equal). **Nadia Matos de Melo Fernandes:** Project administration (supporting). **Sumaira Khalfe:** Project administration (supporting). **Britta Stordal:** Conceptualization (lead); formal analysis (lead); methodology (lead); supervision (lead); writing – original draft (lead).

## FUNDING INFORMATION

No funding to declare.

## CONFLICT OF INTEREST STATEMENT

The authors have no conflicts of interest to declare.

## Supporting information


Tables S1
Table S2
Table S3

## Data Availability

Data is available by contacting the corresponding author

## References

[1] Sung H , Ferlay J , Siegel RL , et al. Global cancer statistics 2020: GLOBOCAN estimates of incidence and mortality worldwide for 36 cancers in 185 countries. CA Cancer J Clin. 2021;71(3):209‐249. doi:10.3322/caac.21660 33538338

[2] Fitch MI , Deane K , Howell D , Gray RE . Women's experiences with ovarian cancer: reflections on being diagnosed. Can Oncol Nurs J. 2002;12(3):152‐159. Accessed August 31, 2022. http://canadianoncologynursingjournal.com/index.php/conj/article/view/406 12271917 10.5737/1181912x123152159

[3] Redman C , Duffy S , Dobson C . Improving early detection of ovarian cancer. Practitioner. 2011;255(1741):27‐30. 21776915

[4] Low EL , Waller J , Menon U , Jones A , Reid F , Simon AE . Ovarian cancer symptom awareness and anticipated time to help‐seeking for symptoms among UK women. J Fam Plann Reprod Health Care. 2013;39(3):163‐171. 23709609 10.1136/jfprhc-2012-100473

[5] Goff BA , Mandel LS , Melancon CH , Muntz HG . Frequency of symptoms of ovarian cancer in women presenting to primary care clinics. JAMA. 2004;291(22):2705‐2712. doi:10.1001/jama.291.22.2705 15187051

[6] Office for National Statistics . Cancer survival in England ‐ adults diagnosed. Published 2019. Accessed August 12, 2022. https://www.ons.gov.uk/peoplepopulationandcommunity/healthandsocialcare/conditionsanddiseases/datasets/cancersurvivalratescancersurvivalinenglandadultsdiagnosed

[7] Zeppernick F , Meinhold‐Heerlein I . The new FIGO staging system for ovarian, fallopian tube, and primary peritoneal cancer. Arch Gynecol Obstet. 2014;290(5):839‐842. doi:10.1007/s00404-014-3364-8 25082067

[8] NICE . Ovarian Cancer: Recognition and Initial Management: Clinical Guideline [CG122]. National Institute for Health and Care Excellence; 2011. 31815390

[9] Cancer Research UK Ovarian Cancer Incidence by Age . Cancer Research UK; 2019. Accessed August 12, 2022. https://www.cancerresearchuk.org/health‐professional/cancer‐statistics/statistics‐by‐cancer‐type/ovarian‐cancer/incidence#heading‐One

[10] Cancer Research UK . Ovarian Cancer Mortality by Age. Cancer Research UK; 2019. Accessed August 12, 2022. https://www.cancerresearchuk.org/health‐professional/cancer‐statistics/statistics‐by‐cancer‐type/ovarian‐cancer/mortality#heading‐One

[11] Sasieni PD , Shelton J , Ormiston‐Smith N , Thomson CS , Silcocks PB . What is the lifetime risk of developing cancer?: the effect of adjusting for multiple primaries. Br J Cancer. 2011;105(3):460‐465. doi:10.1038/bjc.2011.250 21772332 PMC3172907

[12] Estimates of the Population for the UK , England and Wales, Scotland and Northern Ireland ‐ Mid 2017. Office for National Statistics; 2017. Accessed April 4, 2022. https://www.ons.gov.uk/peoplepopulationandcommunity/populationandmigration/populationestimates/datasets/populationestimatesforukenglandandwalesscotlandandnorthernireland

[13] Deaths Registered by Single Year of Age, UK . Office for National Stat; 2017. Accessed April 4, 2022. https://www.ons.gov.uk/peoplepopulationandcommunity/birthsdeathsandmarriages/deaths/datasets/deathregistrationssummarytablesenglandandwalesdeathsbysingleyearofagetables

[14] Lifetime Risk Calculator . Cancer Research UK; 2022. Accessed April 4, 2022. https://www.cancerresearchuk.org/health‐professional/cancer‐statistics/cancer‐stats‐explained/our‐calculations‐explained#heading‐Eight

[15] Trabert B , Tworoger SS , O'Brien KM , et al. The risk of ovarian cancer increases with an increase in the lifetime number of ovulatory cycles: an analysis from the ovarian cancer cohort consortium (OC3). Cancer Res. 2020;80(5):1210‐1218. doi:10.1158/0008-5472.CAN-19-2850 31932455 PMC7056529

[16] Stewart LM , Holman CDJ , Aboagye‐Sarfo P , Finn JC , Preen DB , Hart R . In vitro fertilization, endometriosis, nulliparity and ovarian cancer risk. Gynecol Oncol. 2013;128(2):260‐264. doi:10.1016/j.ygyno.2012.10.023 23116937

[17] Luan NN , Wu QJ , Gong TT , Vogtmann E , Wang YL , Lin B . Breastfeeding and ovarian cancer risk: a meta‐analysis of epidemiologic studies. Am J Clin Nutr. 2013;98(4):1020‐1031. doi:10.3945/ajcn.113.062794 23966430 PMC3778857

[18] Babic A , Sasamoto N , Rosner BA , et al. Association between breastfeeding and ovarian cancer risk. JAMA Oncol. 2020;6(6):e200421. doi:10.1001/jamaoncol.2020.0421 32239218 PMC7118668

[19] Karlsson T , Johansson T , Höglund J , Ek WE , Johansson Å . Time‐dependent effects of oral contraceptive use on breast, ovarian, and endometrial cancers. Cancer Res. 2021;81(4):1153‐1162. doi:10.1158/0008-5472.CAN-20-2476 33334812

[20] Simon AE , Wardle J , Grimmett C , et al. Ovarian and cervical cancer awareness: development of two validated measurement tools. J Fam Plann Reprod Health Care. 2012;38(3):167‐174. 21933805 10.1136/jfprhc-2011-100118 PMC3970720

[21] Office for National Statistics . London Region Local Area Report ‐ 2011 Census. Office for National Statistics; 2011 https://www.nomisweb.co.uk/reports/localarea?compare=E12000007#section_9

[22] Qualtrics XM ‐ The Leading Experience Management Software. Accessed August 5, 2022. https://www.qualtrics.com/

[23] Cancer Research UK . The cancer awareness measures (CAM). Cancer Research UK. Published October 28, 2014. Accessed January 13, 2023. https://www.cancerresearchuk.org/health‐professional/awareness‐and‐prevention/the‐cancer‐awareness‐measures‐cam

[24] Google Maps . Accessed July 29, 2022. https://www.google.co.uk/maps/@51.9683332,‐0.251021,14z

[25] LSOA Atlas ‐ London Datastore . Accessed July 29, 2022. https://data.london.gov.uk/dataset/lsoa‐atlas

[26] Penney B . The English indices of deprivation 2019 (IoD2019). Ministry of Housing, Communities & Local Government 2019. Accessed July 28, 2022. https://assets.publishing.service.gov.uk/government/uploads/system/uploads/attachment_data/file/835115/IoD2019_Statistical_Release.pdf

[27] Minitab 17 Statistical Software . Published online 2010. www.minitab.com

[28] GraphPad . https://www.graphpad.com/

[29] Campaign by raising awareness | Target Ovarian Cancer. Accessed August 31, 2022. https://targetovariancancer.org.uk/get‐involved/campaign/campaigning‐toolkit/campaign‐raising‐awareness

[30] NHS . Help Us Help You ‐ Abdominal and Urological Symptoms of Cancer. Published 2020. Accessed August 2, 2022. https://campaignresources.phe.gov.uk/resources/campaigns/116‐help‐us‐help‐you‐‐‐abdominal‐and‐urological‐symptoms‐of‐cancer/resources?query=

[31] Target Ovarian Cancer . *Pathfinder 2022: Faster*, *Further and Fairer*. Target Ovarian Cancer. 2022. Accessed January 13, 2023. https://targetovariancancer.org.uk/get‐involved/campaign/policy/pathfinder

[32] Fricker S , Galesic M , Tourangeau R , Yan T . An experimental comparison of web and telephone surveys. Public Opin Q. 2005;69(3):370‐392. doi:10.1093/poq/nfi027

[33] Connor K , Hudson B , Power E . Awareness of the signs, symptoms, and risk factors of cancer and the barriers to seeking help in the UK: comparison of survey data collected online and face‐to‐face. JMIR Cancer. 2020;6(1):e14539. doi:10.2196/14539 31951219 PMC6996748

[34] Brain KE , Smits S , Simon AE , et al. Ovarian cancer symptom awareness and anticipated delayed presentation in a population sample. BMC Cancer. 2014;14:171. doi:10.1186/1471-2407-14-171 24612526 PMC3975332

[35] Harlow BL , Cramer DW , Bell DA , Welch WR . Perineal exposure to talc and ovarian cancer risk. Obstet Gynecol. 1992;80(1):19‐26. 1603491

[36] Gertig DM , Hunter DJ , Cramer DW , et al. Prospective study of talc use and ovarian cancer. J Natl Cancer Inst. 2000;92(3):249‐252. doi:10.1093/jnci/92.3.249 10655442

[37] O'Brien KM , Tworoger SS , Harris HR , et al. Association of powder use in the genital area with risk of ovarian cancer. Jama. 2020;323(1):49‐59. doi:10.1001/jama.2019.20079 31910280 PMC6990816

[38] Fallowfield L , Fleissig A , Barrett J , et al. Awareness of ovarian cancer risk factors, beliefs and attitudes towards screening: baseline survey of 21,715 women participating in the UK collaborative trial of ovarian cancer screening. Br J Cancer. 2010;103(4):454‐461. PM:20648018. 20648018 10.1038/sj.bjc.6605809 PMC2939792

[39] Jacobs IJ , Menon U , Ryan A , et al. Ovarian cancer screening and mortality in the UK collaborative trial of ovarian cancer screening (UKCTOCS): a randomised controlled trial. Lancet. 2016;387(10022):945‐956. doi:10.1016/S0140-6736(15)01224-6 26707054 PMC4779792

[40] Denscombe M . Web‐based questionnaires and the mode effect: an evaluation based on completion rates and data contents of near‐identical questionnaires delivered in different modes. Soc Sci Comput Rev. 2006;24(2):246‐254. doi:10.1177/0894439305284522

[41] Denscombe M . The length of responses to open‐ended questions: a comparison of online and paper questionnaires in terms of a mode effect. Soc Sci Comput Rev. 2008;26(3):359‐368. doi:10.1177/0894439307309671

[42] Bandera EV , Lee VS , Qin B , Rodriguez‐Rodriguez L , Powell CB , Kushi LH . Impact of body mass index on ovarian cancer survival varies by stage. Br J Cancer. 2017;117(2):282‐289. doi:10.1038/bjc.2017.162 28588323 PMC5520512

[43] Gică C , Cigăran RG , Botezatu R , et al. Secondary amenorrhea and infertility due to an inhibin B producing granulosa cell tumor of the ovary. A Rare Case Report and Literature Review. Med Kaunas Lith. 2021;57(8):829. doi:10.3390/medicina57080829 PMC8398809 34441035

[44] Littrell LA , Inwards CY , Hazard FK , Wenger DE . Juvenile granulosa cell tumor associated with Ollier disease. Skeletal Radiol. Published online March 16, 2022. 2023;52(3):605‐612. doi:10.1007/s00256-022-04033-5 35296906

[45] Tavani A , Negri E , Franceschi S , Parazzini F , La Vecchia C . Risk factors for epithelial ovarian cancer in women under age 45. Eur J Cancer. 1993;29A(9):1297‐1301. doi:10.1016/0959-8049(93)90077-s 8343272

[46] Cirillo PM , Wang ET , Cedars MI , Chen LM , Cohn BA . Irregular menses predicts ovarian cancer: prospective evidence from the child health and development studies. Int J Cancer. 2016;139(5):1009‐1017. doi:10.1002/ijc.30144 27082375 PMC6917033

[47] Rizzuto I , Behrens RF , Smith LA . Risk of ovarian cancer in women treated with ovarian stimulating drugs for infertility. Cochrane Database Syst Rev. 2019;6:CD008215. doi:10.1002/14651858.CD008215.pub3 31207666 PMC6579663

[48] van Leeuwen FE , Klip H , Mooij TM , et al. Risk of borderline and invasive ovarian tumours after ovarian stimulation for in vitro fertilization in a large Dutch cohort. Hum Reprod Oxf Engl. 2011;26(12):3456‐3465. doi:10.1093/humrep/der322 PMC3212878 22031719

[49] Källén B , Finnström O , Nygren KG , Otterblad Olausson P , Wennerholm UB . In vitro fertilisation in Sweden: obstetric characteristics, maternal morbidity and mortality. BJOG. 2005;112(11):1529‐1535. doi:10.1111/j.1471-0528.2005.00745.x 16225574

[50] Varela‐Rey M , Woodhoo A , Martinez‐Chantar ML , Mato JM , Lu SC . Alcohol, DNA methylation, and cancer. Alcohol Res Curr Rev. 2013;35(1):25‐35. 10.35946/arcr.v35.1.04 PMC3860423 24313162

[51] Rumgay H , Murphy N , Ferrari P , Soerjomataram I . Alcohol and cancer: epidemiology and biological mechanisms. Nutrients. 2021;13(9):3173. doi:10.3390/nu13093173 34579050 PMC8470184

[52] Rota M , Pasquali E , Scotti L , et al. Alcohol drinking and epithelial ovarian cancer risk. A systematic review and meta‐analysis. Gynecol Oncol. 2012;125(3):758‐763. doi:10.1016/j.ygyno.2012.03.031 22449732

[53] Liu Z , Zhang TT , Zhao JJ , et al. The association between overweight, obesity and ovarian cancer: a meta‐analysis. Jpn J Clin Oncol. 2015;45(12):1107‐1115. doi:10.1093/jjco/hyv150 26491203

[54] Jones SC , Magee CA , Francis J , et al. Australian women's awareness of ovarian cancer symptoms, risk and protective factors, and estimates of own risk. Cancer Causes Control. 2010;21(12):2231‐2239. doi:10.1007/s10552-010-9643-1 20865449

[55] Lockwood‐Rayermann S , Donovan HS , Rambo D , Kuo CWJ . Women's awareness of ovarian cancer risks and symptoms. Am J Nurs. 2009;109(9):36‐45. doi:10.1097/01.NAJ.0000360309.08701.73 19704232

[56] Chelimo C , Wouldes TA , Cameron LD , Elwood JM . Risk factors for and prevention of human papillomaviruses (HPV), genital warts and cervical cancer. J Infect. 2013;66(3):207‐217. doi:10.1016/j.jinf.2012.10.024 23103285

[57] Puckett MC , Townsend JS , Gelb CA , Hager P , Conlon A , Stewart SL . Ovarian cancer knowledge in women and providers following education with inside knowledge campaign materials. J Cancer Educ. 2018;33(6):1285‐1293. doi:10.1007/s13187-017-1245-0 28646458 PMC5742303

[58] David G , Mary S . In: Acheson SD , ed. Inequalities in Health: the Evidence Presented to the Independent Inquiry into Inequalities in Health. Chaired by. Policy Press; 1999.

[59] Robb K , Stubbings S , Ramirez A , et al. Public awareness of cancer in Britain: a population‐based survey of adults. Br J Cancer. 2009;101(Suppl 2):S18‐S23. doi:10.1038/sj.bjc.6605386 PMC2790705 19956158

[60] Gathani T , Balkwill A , Moser KA , Reeves GK , Green J , Beral V . Incidence of ovarian and endometrial cancer by ethnicity in the million women study. Int J Epidemiol. 2015;44(suppl_1):i198. doi:10.1093/ije/dyv096.316

[61] Morris CR , Sands MT , Smith LH . Ovarian cancer: predictors of early‐stage diagnosis. Cancer Causes Control CCC. 2010;21(8):1203‐1211. doi:10.1007/s10552-010-9547-0 20364367

[62] Marlow LAV , Waller J , Wardle J . Barriers to cervical cancer screening among ethnic minority women: a qualitative study. J Fam Plann Reprod Health Care. 2015;41(4):248‐254. doi:10.1136/jfprhc-2014-101082 25583124 PMC4621371

[63] Webb R , Richardson J , Esmail A , Pickles A . Uptake for cervical screening by ethnicity and place‐of‐birth: a population‐based cross‐sectional study. J Public Health Oxf Engl. 2004;26(3):293‐296. doi:10.1093/pubmed/fdh128 15454600

