# Supplementary material for: Awareness of ovarian cancer symptoms and risk factors in a young ethnically diverse British population
Source: Cancer Med. 2023 Feb 7;12(8):9879–92. doi: 10.1002/cam4.5670 (PMC10166982; doi:10.1002/cam4.5670)
Supplement: Supplementary file 1 — Tables S1 Table S2 Table S3 [file CAM4-12-9879-s001.docx]

**Table 1** – Study Participants

|  |  | All Participants  (n = 449) | Age  < 30 | Age  ≥ 30 | P-Value  Chi Squared vs Age Groups |
| --- | --- | --- | --- | --- | --- |
| Age  (n = 420) |  | Mean 27.89 ± 11.44  Range 18 -75 years | 312 (74.2%) | 108 (25.7%) |  |
| Gender  (n = 449) | Female | 337 (75.1%) | 231 (73.6%) | 92 (85.2%) | 0.045 |
|  | Male | 108 (24.1%) | 80 (25.5%) | 15 (13.9%) |  |
|  | Prefer not to say | 4 (0.8%) | 3 (1.0%) | 1 (0.9%) |  |
| Ethnicity  (n = 427) | White | 172 (40.3%) | 84 (26.8%) | 85 (78.7%) | 0.000 |
|  | Asian/Asian British | 125 (29.3%) | 114 (36.4%) | 10 (9.3%) |  |
|  | Black/African/Caribbean/Black British | 77 (18.0%) | 70 (22.4%) | 6 (5.6%) |  |
|  | Mixed/Multiple Ethnic Groups | 21 (4.9%) | 16 (5.1%) | 4 (3.7%) |  |
|  | Other | 26 (6.1%) | 23 (7.3%) | 3 (2.8%) |  |
|  | Prefer not to say | 6 (1.4%) | 6 (1.9%) | 0 (0.0%) |  |
| Postcode  (n = 355) | London Region | 276 (77.7%) | 229 (84.5%) | 47 (56.0%) | 0.000 |
|  | Non-London Region | 79 (22.3%) | 42 (15.5%) | 37 (44.0%) |  |
| Deprivation  (n = 355) | High Deprivation (Decile 1-5) | 222 (62.5%) | 191 (70.5%) | 31 (36.9%) | 0.000 |
|  | Low Deprivation (Decile 6-10) | 133 (37.5%) | 80 (29.5%) | 53 (63.1%) |  |
| Education  (n = 421) | Completed University Education | 257 (62.0%) | 165 (47.5%) | 92 (85.2%) | 0.000 |
|  | Completed School Education | 165 (35.2%) | 149 (52.6%) | 16 (14.8%) |  |
| Cancer Diagnosis  (n = 412) | Cancer Diagnosis | 43 (10.4%) | 11 (3.6%) | 32 (30.5%) | 0.000 |
|  | No Cancer Diagnosis | 363 (88.1%) | 291 (94.8%) | 72 (68.6%) |  |
|  | Prefer not to say | 6 (1.5%) | 5 (1.5%) | 1 (1.0%) |  |
| Cancer Experience  (n = 416) | Personal Experience of Cancer | 291 (70.0%) | 193 (62.5%) | 98 (91.6%) | 0.000 |
|  | No Experience of Cancer | 125 (30.0%) | 116 (37.5%) | 9 (8.4%) |  |

**Table S1** – Answers Scored Correctly for Each Symptom and Risk Factor

| **Symptom** | **Included as Correct Answer** |
| --- | --- |
| Persistent Abdominal Pain | Abdominal pain, tummy pain, stomach pain, stomach ache, abdominal discomfort |
| Persistent Pelvic Pain | Pelvic pain, pelvis pain, pelvic pressure, pelvic discomfort |
| Persistent Bloating | Bloating, bloatiness, bloated abdomen, bloating of stomach |
| Increased Abdominal Size | Swollen tummy, abdominal swelling |
| Feeling Full Persistently | Feeling full, feeling full quickly |
| Difficulty Eating | Difficult eating, |
| Passing more Urine | Increase in urinary frequency, need to pee more often, constant urination, |
| Change in Bowel Habit | Constipation, diarrhoea |
| Extreme Fatigue | Fatigue, tiredness, exhaustion |
| Back Pain | Lower back pain, |
| **Risk Factor** | **Included as Correct Answer** |
| Close relative with Ovarian Cancer | Family history, hereditary, inheritance, genetics, genes, BRCA, inherited gene mutation, DNA mutation |
| Past History of Breast Cancer | Breast Cancer |
| Hormone Replacement Therapy | HRT, hormonal replacement |
| Overweight (BMI over 25) | Overweight, obesity, obese, high weight |
| Endometriosis | *No additional terms* |
| Ovarian Cysts | Polycystic ovary syndrome, PCOS |
| Over 50 Years Old | Age, ageing, getting older, old age, increasing age |
| Talcum Powder in Genital Area | Using talcum powder |
| Having IVF Treatment | Fertility treatment, fertility drugs, |
| Not having children | Nulliparous, lack of pregnancy, no childbirth |
| Gone through Menopause | Menopause |
| Being a Smoker | Smoking |

**Table S2** – Answers Included for Analysis of Incorrect Symptoms and Risk Factors

| **Incorrect Symptom** | **Included for Analysis** |
| --- | --- |
| Vaginal Spotting / Discharge | Off colour discharge, strange discharge, abnormal discharge, bleeding between periods |
| Pain During Intercourse | Sexual discomfort, pain during sex, bleeding with intercourse |
| Weight Loss | Unexplained weight loss, sudden weight loss, loss of weight |
| Irregular Periods | Irregular menstruation cycles |
| Loss of Appetite | Lack of appetite |
| Nausea | *No additional terms* |
| Headache | *No additional terms* |
| Infertility | Inability to conceive, unable to become pregnant, difficulty getting pregnant |
| **Incorrect Risk Factor** | **Included for Analysis** |
| Multiple Partners | Many sexual partners, early age sexual activity, too much sex |
| STD / Unprotected Sex | Sexual transmission, not using condoms |
| Alcohol | Drinking |
| Poor Diet | Diet, unhealthy diet, |
| Exercise | Physical activity, how active the person is, sedentary life style |
| HPV | *No additional terms* |
| Lack of Smear Test | No Screening |
| Stress | Psychological Stress |
| Contraceptive Pill | Contraceptives |
| Radiation | *No additional terms* |
| Chemicals / Carcinogens | Pollution, pollutants |

**Table S3** – Collapsed Ethnicity Categories

| White | White British, White Irish, Any other White background |
| --- | --- |
| Asian | Indian, Pakistani, Bangladeshi, Chinese, Any other Asian background |
| Black | Black Caribbean, Black African, Any other Black background |
